# On the instability of the giant direct magnetocaloric effect in CoMn_0.915_Fe_0.085_Ge at. % metamagnetic compounds

**DOI:** 10.1038/s41598-020-71149-w

**Published:** 2020-08-26

**Authors:** N. M. Bruno, S. Yuce

**Affiliations:** 1 Department of Mechanical Engineering, South Dakota School of Mines and Technology, Rapid City, SD 57701 USA; 2 Department of Physics, Science and Literature Faculty, Ondokuz Mayis University, Kurupelit, 55139 Samsun, Turkey

**Keywords:** Materials science, Condensed-matter physics, Materials for devices

## Abstract

The giant magnetocaloric effect was quantified in CoMn_1-x_Fe_x_Ge (x = 0.085–0.12) nom. at. % polycrystals across the high temperature hexagonal (P6_3_/mmc) to low temperature orthorhombic (Pnma) phase transition via differential scanning calorimetry (DSC) and multiple (thermo) magnetization measurements. It was found that increasing Fe content led to the decrease of both the martensitic transformation temperature and entropy change ($\Delta S$) at the point of the phase transition. Moreover, first-time magnetocaloric measurements resulted in irreproducible entropy change versus temperature diagrams, which was attributed to the release of internal pressure in bulk samples that disintegrated into powder upon transformation. CoMn_1-x_Fe_x_Ge demonstrated larger magnetic field-induced entropy changes and giant magnetocaloric effect (MCE) compared to other CoMnGe alloys doped with Si, Sn, Ti, and Ga. However, the observed brittleness and apparent change in volume at the magnetic transition was posited to influence the material’s potential for regenerative applications.

## Introduction

### Giant magnetocaloric effects

A caloric effect refers to the adiabatic temperature ($\Delta {T}_{ad})$ or isothermal entropy change $(\Delta {S}_{iso})$ that results from applying an external stimulus to a solid material^1^. These effects can be driven by a magnetic field, $H$, hydrostatic pressure, $P$, mechanical stress, $\sigma$, and an electric potential, $E$, producing a magnetocaloric, barocaloric, elastocaloric (or mechanocaloric), and electrocaloric effect, respectively^1^. Among them, the most studied is the magnetocaloric effect (MCE), but more recently the multiphysical coupling in some active materials has prompted interest in caloric effects driven by combined stimuli, namely the multicaloric effects^2–6^. In multicaloric alloys, the magnetocaloric behavior is coupled to an additional driving mechanism, such as hydrostatic pressure.

Materials that undergo magnetic transitions around room temperature are candidates for conventional MCE applications, where the $\Delta {S}_{iso}$ and $\Delta {T}_{ad}$ are largest around the magnetic Curie point, ${T}_{Curie}$^7^. Conversely, in giant MCE materials, the magnetic transition occurs simultaneously with a first-order structural change in the lattice. These structural (crystallographic) changes can include dilatation or transformation^8–10^. Thus, the resulting MCE is widely understood to combine with the structural transition latent heat.

Some of the most common materials studied for their magnetostructural giant MCE are GdSiGe^11^, Fe_2_P compounds^12^, and NiMn-based intermetallic Heusler alloys, namely metamagnetic shape memory alloys (MMSMAs)^13–16^. In NiMn-based MMSMAs, a first-order reversible and diffusionless martensitic transformation can occur while sweeping the temperature. Above critical transformation temperatures, the MMSMA is typically characterized by a cubic and ferromagnetic austenite phase, and below them, a non-magnetic non-cubic martensite phase. During the martensitic transformation, macroscopic strain^3,17–19^, crystallographic volume^20,21^, bulk magnetization^22,23^, and electric resistivity^24^ are all subject to large fluctuations. The changes observed in these physical properties are explained through free-energy analysis and equilibrium thermodynamics.

Most importantly, the crystallographic transformation in NiMn-based MMSMAs can be driven by applying a magnetic field to the martensite phase. As such, the magnetocaloric effect is expected to produce heat (exothermic reaction) through magnetizing the individual martensite and austenite phases, and cooling (endothermic reaction) from the martensite-to-austenite latent heat^25–30^. These two opposing entropic contributions are viewed, here, as a main limiting factor hindering the applicability of NiMn-based MMSMAs in MCE applications. The authors believe a more practical material should exhibit a first-order magnetostructural transition around a ferromagnetic low temperature martensite phase and a non-magnetic high temperature austenite phase.

### Magnetostructural transformations in CoMnGe

In recent years, TT’X (T, T’ = transition metals, X = Si, Ge, Sn) alloys^31–33^ have attracted much attention due to their remarkable giant magnetocaloric effects^34–43^. One of these compounds, namely CoMnGe, exhibits a first-order transition around ferromagnetic martensite and non-magnetic (non-cubic) austenite. According to previous studies, CoMnGe can exhibit a reversible structural phase transition between ferromagnetic TiNiSi-type orthorhombic (Pnma) martensite and a paramagnetic Ni_2_In-type hexagonal (P6_3_/mmc) austenite. The phase transition has been reported to be reversible and diffusionless^44–51^. In these alloys, the (martensitic) transformation temperatures can vary between 420 K and 650 K depending on the alloy’s composition and prior thermal treatment^31,44^. It is apparent that thermal treatments influence the lattice site occupancy of Mn and Co atoms, i.e., long-range crystallographic order^32,47^. Long-range order has also been widely reported to influence the bulk saturation magnetization in CoMnGe^32,47^ and transformation temperature span, thermal hysteresis, and magnetic field sensitivity in other metamagnetic materials^23,43,44,52^.

CoMnGe is a unique MMSMA because, unlike NiMn-based alloys, the austenite phase takes on the Ni_2_In-type hexagonal structure^32^. Moreover, it has been well documented that the compound exhibits a volume expansion of nearly 4%^47,53,54^ during the austenite to orthorhombic martensite (forward) transformation. When the CoMnGe compound has been quenched in bulk form, the first martensitic transformation almost always results in microstructural crack propagation and disintegration from bulk polycrystals to a fine grain powder^55–60^. The authors believe this is caused by a combination of Bain path transformation physics inherent to the hexagonal to orthorhombic transformation, the simultaneous large negative thermal expansion and volume change, and the few available slip systems in the hexagonal phase, but more work is needed to definitively determine why bulk samples decompose while transforming. Nonetheless, the change in sample structure is linked to a change in internal hydrostatic pressure, which influences the transformation characteristics, such as transformation temperatures and entropy change (latent heat)^55–57^. This pressure sensitivity clearly demonstrates that CoMnGe compounds are truly multicaloric in nature and couple magnetic, thermal, and mechanical energy domains.

#### Hydrostatic pressure sensitivity in CoMnGe

It has been reported that quenching CoMnGe alloys from high temperatures can decrease martensitic transformation temperatures compared to slow cooling and stabilize the austenite phase^56^, because a high internal pressure is produced during rapid cooling. This internal pressure has been linked to the softening of acoustic phonon modes and ultimately covalent bonding, which affects the thermodynamic state of the crystal lattice^55^. Moreover, lattice parameters are influenced by quench rate^56^. Tempering, or relieving internal pressure with secondary annealing treatments, has been shown to recover (increase) transformation temperatures in CoMnGe compounds where the critical temperatures were lower following the original quench^56^.

Interestingly, the transformation temperature–pressure dependence of CoMnGe is opposite to that of NiMn-based MMSMAs, presumably due to the compound’s negative thermal expansion coefficient^55^. Indeed, the magnetic field—transformation temperature proportionality is positive in CoMnGe, but pressure—temperature proportionality is reportedly negative^61^, suggesting that a giant inverse elastocaloric, or barocaloric effect, may be possible in the compound. However, due to thermodynamic limitations and the physics of the phase transformation, the stress-induced transformation would need to take place between the martensite to austenite phase and overcome the compound’s inherent brittleness.

A brief literature review demonstrates that the pressure sensitivity in CoMnGe drives much of its development. For instance, internal pressure can be controlled by thermal quenching, and also on the atomic level via chemical doping (producing what is referred to as “chemical pressure”^44,54,61^). It has been shown that replacing Fe for Mn in Co(Mn,Fe)Ge results in a decrease in lattice volume, stabilizes the hexagonal phase^44,54^, and decreases transformation temperatures. Moreover, substituting Fe for Mn has been shown to be more effective at reducing transformation temperatures than substituting Fe for another element, such as Co^54^.

Several groups have investigated the change in CoMnGe transformation characteristics with chemical doping. Most studies aim to decrease transformation temperatures to achieve near-room temperature transitions and retain favorable magnetocaloric properties, or to tune the ferromagnetic transition of martensite to coincide with the structural one. Replacing Co, Mn, or Ge with a fourth element, e.g. Ga for Ge^62^, V for Mn^53^, Fe for Co^63^, Sn for Ge^41^, and Ti for Mn^64^, almost always decreases transformation temperatures by changing the volume of the hexagonal phase. Moreover, the magnetic state of the orthorhombic phase has been posited to depend on dopant valency^53^. Therefore, the transition temperature can be reduced for a near room-temperature magnetocaloric effect, and the magnetic Zeeman energy can be simultaneously increased.

#### Magnetism in CoMnGe

Early works^47^ on the CoMnGe compound investigated the effect of reducing Mn concentration for equal parts of Co and Ge on the magnetic spins. A decrease in the magnetic moment from 3.86 ${\mu }_{B}$/f.u. in stoichiometric CoMnGe, to 2.58 ${\mu }_{B}$/f.u. in CoMn_0.95_Ge was observed suggesting Mn–Mn interaction significantly contributed to the bulk magnetic saturation. In further works, it was determined that primarily Co and Mn contribute to the magnetic spin of Co_0.95_MnGe_1.14_ at. % where the Mn site exhibited 3.08 ${\mu }_{B}$, the Co site contributed a mere 0.64 ${\mu }_{B}$^42^ and the Ge site contributed only $-0.11 {\mu }_{B}$^36^. It was concluded that Mn carried a much larger moment than Co; therefore, CoMnGe compounds that are rich in Co in exchange for Mn exhibit a lower, though not significantly, saturation magnetization^56^. Subsequent studies have attempted to increase saturation magnetization of the bulk compound by 1. changing the spin coupling on the Ge site^41,55,62^; 2. increasing the spin-coupling on the Mn site^44,53,55,64^; and 3. increasing the spin coupling on the Co site^55,57,63^, each of which inadvertently changed the volume of the hexagonal phase and decreased the transformation temperatures.

Of the three methods of controlling magnetic spin, a brief review of the available literature clearly demonstrates that specifically replacing Mn for another element offers the greatest potential to increase saturation magnetization, and, in effect, the MCE. It was shown in Ref.^55^ that CoMn_0.97_In_0.03_Ge exhibited a saturation magnetization of 10 emu g^−1^ larger than near stoichiometric CoMnGe, i.e. 85 emu g^−1^ rather than 75 emu g^−1^. In CoMnTiGe and CoMnVGe alloys, saturation magnetization around room temperature was measured to be nearly 80 emu g^−1^^53,62,64^. In Ref.^44^, we showed that CoMn_0.915_Fe_0.085_Ge could exhibit a saturation magnetization of nearly 95 emu g^−1^ at 200 K, suggesting than Fe enhances the magnetic spin on the Mn site. Conversely, replacing 5% Ge with Sn or Ga has been shown to increase saturation magnetization in the bulk CoMnGe compound by only 5 emu g^−1^^41,62^.

#### Magnetocaloric effect in CoMnGe

Aside from the tunability of the transformation temperatures, the magnetic Zeeman energy and saturation magnetization of CoMnGe offers significant advantages for MCE applications compared to other MMSMAs. For instance, the metamagnetic transition occurs over a small temperature range, and the hexagonal to orthorhombic structural transition exhibits small thermal hysteresis ($\approx$ 10 K^42,44,53,62^), each of which aids in the reduction in the required magnetic field to drive the magnetostructural transition and MCE^18,65–67^. Moreover, with a ferromagnetic martensite phase, the CoMnGe compounds and its doped alloys will exhibit a direct giant magnetocaloric effect via adiabatic demagnetization from field-stabilized martensite. The entropy change has been reported to be larger than typical NiMn-based compounds^68^ presumably due to the reversal of the entropic contributions in CoMnGe, mentioned above. Under an applied field of 10 kOe, entropy changes in Co_0.95_MnGe_1.14_^42^, CoMn_(1-x)_Ti_x_Ge^64^, and CoMnGe_1-x_Sn_x_^41^, have been reported to be between 5.5 J kgK^−1^ and 6.5 J kgK^−1^ at temperatures that range from 280 to 330 K. Under 50 kOe, Co_x_Fe_1-x_MnGe, Co_1.09_Mn_0.91_Ge, and (FeNi)MnGe, have reportedly produced 9 J kgK^−1^^63^, 35 J kgK^−1^^56^, and 60 J kgK^−1^^57^, respectively; however, the latter appears to be an over-approximation. Due to the characteristics of the martensitic transition in CoMnGe based compounds, 50 kOe is enough to capture the entire transformation latent heat, as 70 kOe only marginally increases the entropy change. Applying and removing 70 kOe has been reported to produce entropy changes of 35 J kgK^−1^ and 30 J kgK^−1^ in CoMnGe_0.95_Ga_0.05_^62^ and CoMn_0.95_V_0.05_Ge^53^, respectively, at temperatures of 310 K and 260 K.

Despite the favorable characteristics of the martensitic transformation in CoMnGe, the sensitivity of the critical transformation temperature to hydrostatic pressure may hinder the compound’s use in MCE applications. Here, we demonstrate that the magnetocaloric entropy change is susceptible to cyclic transformation instability. First, we replace Mn with Fe to simultaneously increase the magnetic moment of the Mn site and to decrease the transformation temperatures from that of stoichiometric CoMnGe, while maintaining low thermal hysteresis. We subsequently investigate the effect of cyclic stability and compound physical state on the magnetocaloric measurement protocol. For instance, bulk as quenched samples are observed to exhibit lower transformation temperatures from built-up microstructural pressure, as mentioned above. Upon transforming between austenite to martensite, burst-type transformations are observed in isothermal magnetization measurements, which resulted in spurious entropy changes quantified by post-processing data from maiden transformation cycles^69^; upon transforming, bulk samples had disintegrated to powder due to their negative thermal expansion characteristics, thus releasing microstructural pressure, and exhibited higher, more stable, transformation temperatures, characteristics, and giant MCE.

## Experimental and data processing procedures

### Synthesis and annealing

CoMn_1-x_Fe_x_Ge (x = 0, 0.085, 0.0975, 0.1, 0.11, and 0.12) specimens were prepared by arc-melting high purity elements Co (99.999%), Mn (99%), Fe (99.99%), and Ge (99.9999%) in argon atmosphere with a liquid-cooled copper mold. Ingots were flipped and re-melted five times to ensure homogeneity^39^. The cooling rates of arc-melted buttons were uncontrolled. Pre- and post-melt mass measurements indicated a Mn loss of less than 2 wt. % during melting. Ingots were then annealed in quartz ampoules that were evacuated and backfilled under 0.5 Torr of high purity argon. Annealing (homogenization) treatments were performed at 1,023 K for 60 h, whereby the samples were slow cooled at 5 K min^−1^ to 643 K to promote Co-Mn ordered site occupancy, followed by uncontrolled cooling to room temperature.

The compound compositions were measured using a CAMECA SX-50 scanning electron microprobe equipped with lithium fluoride (LIF), thallium acid phthalate (TAP), and pentaerythritol (PET) diffracting crystals for wavelength dispersive spectroscopy (WDS). After checking spectra interferences, Ge concentration was measured using the ${L}_{\alpha }$ energy with the TAP crystal and Co, Mn, and Fe concentrations using ${K}_{\alpha }$ energies with the LIF crystal. For composition analysis, a beam current of 20 nA under 15 kV was employed for nearly 1.5 min. The microprobe was also equipped with a backscatter electron (BSE) detector, which was employed for imaging the samples in their annealed bulk states.

### Differential scanning calorimetry (DSC)

The martensitic transformation temperatures and transition latent heat were measured by interpreting thermograms from differential scanning calorimetry (DSC) on virgin annealed samples. Samples with a mass greater than 10 mg were measured in aluminum pans with a heating/cooling rate of 10 K min^−1^. To compute the latent heat of the transition, a sapphire standard was first exposed to the same DSC procedure prior to the run and used to calibrate the heat-flow signatures. At least two thermal cycles were performed on each composition, both of which started at low temperatures (i.e. in the martensite phase), in an attempt to observe differences on subsequent runs and reveal cyclic transformation instability. However, we later found that since the DSC measurements started in the martensite phase, some samples had released internal pressure during the maiden transformation from room temperature austenite to low temperature martensite.

### Thermo-magnetization measurements (MT)

Thermo-magnetic and isothermal magnetization measurements were performed using a Quantum Design MPMS3 superconducting quantum interference device (SQUID) vibrating sample magnetometer (VSM). The same samples that were first subjected to DSC were heated to 400 K from room temperature under zero magnetic field. Once stabilizing at 400 K, a magnetic field was applied, and the sample was cooled at 5 K min^−1^ to 10 K while magnetization was measured. After stabilizing at 10 K, the sample was again heated in field to 400 K at the same rate and magnetization was measured. The field was then increased, and the process was repeated. The heating and cooling protocols used, herein, are referred to as field-heating (FH) and field-cooling (FC), respectively^70^.

### Isothermal magnetization (MH) measurements No. 1

Once the composition that exhibited the best giant MCE properties was selected, isothermal magnetization measurements were further performed on virgin bulk polycrystalline samples to quantify the $\Delta S-T$ diagrams. A continuous cooling protocol, analogous to the continuous heating protocol developed in Refs.^67,70–72^ was employed to ensure the material started each magnetization cycle in the non-magnetic austenite phase. However, data obtained with this method has been reported to be sensitive to transition temperature width and other microstructural features^73^. If the magnetostructural transition width is too sharp, a false magnitude $\Delta S$ can be found^74,75^. The continuous cooling protocol proceed as follows: 1. the sample was heated to 400 K under zero applied magnetic field; 2. the sample temperature was reduced at 10 K min^−1^ to the desired setpoint in the austenite phase; 3. the temperature was stabilized with no undershoot; 4. the magnetic field was ramped at 50 Oe s^−1^ to 70 kOe and magnetization was measured; 5. the field was ramped from 70 kOe to zero and magnetization was measured; and 6. the sample temperature was decreased to the next setpoint. The isothermal magnetization measurements were made in increments of 3 K across a temperature range identified by DSC (335 K to 285 K) to coincide with the martensitic transformation. After the test was completed, the specimen was removed from the VSM; however, the bulk polycrystal had decomposed into a powder upon removal.

### Isothermal magnetization (MH) measurements No. 2

After reviewing post-processed data from the first set of isothermal magnetization measurements, we believed spurious peaks were measured as a result of overlarge temperature increments (3 K) between measurements^67^. Therefore, the continuous cooling experiment was performed again, as described above, with smaller temperature increments of 1 K. Moreover, in the first experimental run, we found the largest entropy change to correspond to a temperature nearly 20 K less than what was measured from DSC peaks. Thus, we performed a second set of measurements on the original sample that had decomposed into powder to investigate the stability in transformation characteristics and quantify any differences between the first and second set of magnetization data. Any measurable changes were attributed to a change in the thermodynamic state of the material thought to be from the release of microstructural pressure and the breakdown from a bulk polycrystal to powder during the first MH run.

### Post-processing MH data to compute the giant MCE

The giant MCE ($\Delta S-T$) can be computed from either isofield thermo-magnetic or isothermal magnetization data. Typically, the Clausius-Clapeyron (CC) equation is appropriate to identify entropy changes, or MCE, that result from an isothermal martensitic transformation^18^. The CC equation is useful for quantifying the entropy difference between two phases at a single temperature and is derived from a thermodynamic free energy potential. In this case, the entropy change across an isobaric and isothermal transformation can be well-defined from the Gibbs free energy as $-\Delta S=\Delta M\frac{dH}{dT}$ where $\Delta M$ is the magnetization change across the structural transformation, and $\frac{dH}{dT}$ is the transformation temperature, $T$, sensitivity to the applied magnetic field, $H$. $\frac{dH}{dT}$ is also referred to as the slope of the two-phase co-existence line on an applied field- critical temperature phase diagram, or CC slope. Typically, isofield thermo-magnetic data is employed to compute $\Delta S$ using the CC equation.

Since the CC equation is only useful at a single temperature, a discretized form of the Maxwell relation and a computational algorithm is typically employed in studies pertaining to the giant MCE. The Maxwell relationship enables the construction of $\Delta S-T$ diagrams and quantification of the refrigerant capacity (RC)^8^ across a temperature interval. There has been much debate in the literature on the applicability of employing the Maxwell relationship to first-order magnetostructural transformations, as it is typically derived under the assumption a single structural phase exhibits a second-order magnetic transition. In magnetostructural transitions, however, the metamagnetic transformation is classified as first-order. Here, we acknowledge the large body of discussions surrounding this topic, but we employ the Maxwell relationship to demonstrate the effect of pressure sensitivity and instability in CoMnGe alloys.

The Maxwell relationship is derived in numerous works as^1^

1 $$\Delta S\left({T}_{K},0\to H\right)=\frac{1}{\Delta {T}_{k}}\left[\underset{0}{\overset{H}{\int }}{M}_{k+1}dH-\underset{0}{\overset{H}{\int }}{M}_{k}dH\right]$$

where $\Delta {T}_{k}={T}_{k+1}-{T}_{k}$, ${T}_{K}=({T}_{k+1}+{T}_{k})/2$, ${T}_{k}$ is the ${k}^{th}$ temperature at which magnetization, ${M}_{k}$, is measured, and $\underset{0}{\overset{H}{\int }}{M}_{k}dH$ is the integral of the isothermal magnetization loop from a zero applied field to $H$ at ${T}_{k}$. Experimental data obtained from isothermal magnetization loops with procedures described, above, were used in conjunction with Eq. (1) to compute $\Delta S-T$ diagrams, herein.

## Results and discussion

### Microstructure and composition

**Figure 1:**
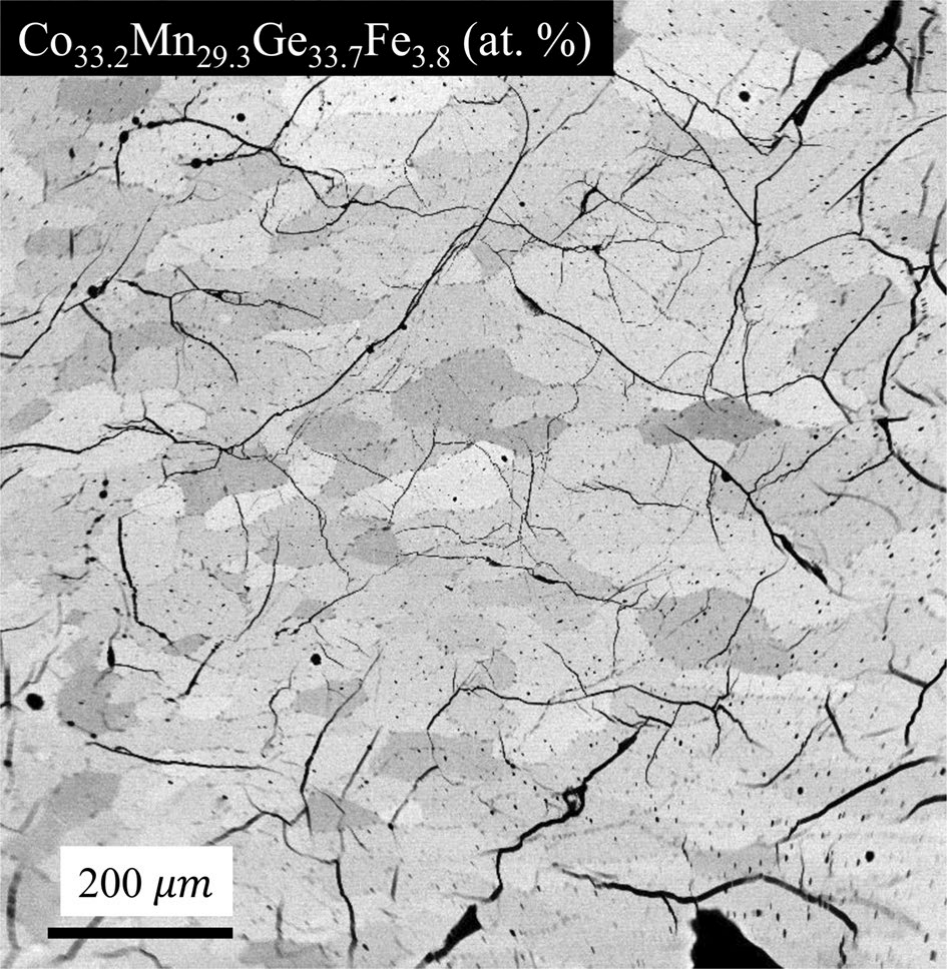
Transgranular fracture in brittle annealed Co_33.2_Mn_29.3_Ge_33.7_Fe_3.8_ at. % (x = 0.12) bulk polycrystals resulting from mechanical polishing and the uncontrolled cooling rate that followed the annealing treatment. Small precipitates were sometimes observed at the grain boundaries after the 60 h annealing treatment with an average composition (measured from four locations) of Co_43.42_Mn_24.60_Ge_25.53_Fe_6.46_ at. %.

Figure 1 depicts the typical microstructure of annealed CoMn_1-x_Fe_x_Ge (x = 0.085–0.12) at. % polycrystals at room temperature. The specimen in Fig. 1 was not subjected to heating or cooling protocols across the martensitic transition prior to microscopy. Nonetheless, transgranular fracture was observed suggesting that either the light pressure applied during mechanical polishing, or the uncontrolled cooling rate following annealing was enough to damage the microstructure. Crack propagation through grains indicates that CoMnGeFe alloys are extremely brittle and exhibit low fracture toughness.

WDS analysis was performed on three grains in each CoMn_1-x_Fe_x_Ge (x = 0.085–0.12) at. % specimen and the compositions were determined to be Co_33.19_Mn_30.46_Ge_33.55_Fe_2.80_ (x = 0.085), Co_33.06_Mn_30.08_Ge_33.66_Fe_3.2_ (x = 0.0975), Co_32.89_Mn_30.02_Ge_33.58_Fe_3.51_ (x = 0.1), Co_33.25_Mn_29.39_Ge_33.79_Fe_3.56_ (x = 0.11), and Co_33.20_Mn_29.30_Ge_33.69_Fe_3.81_ (x = 0.12), respectively. Some samples were observed with martensite twins that were oriented transverse to columnar grain widths. Moreover, small precipitates at grain boundaries were observed in all specimen containing more than 3 at. % Fe. The composition of Fe-rich precipitates was measured to be Co_43.42_Mn_24.60_Ge_25.53_Fe_6.46_ (at. %), however, these precipitates were not present in x = 0.085.

### Differential scanning calorimetry

**Figure 2:**
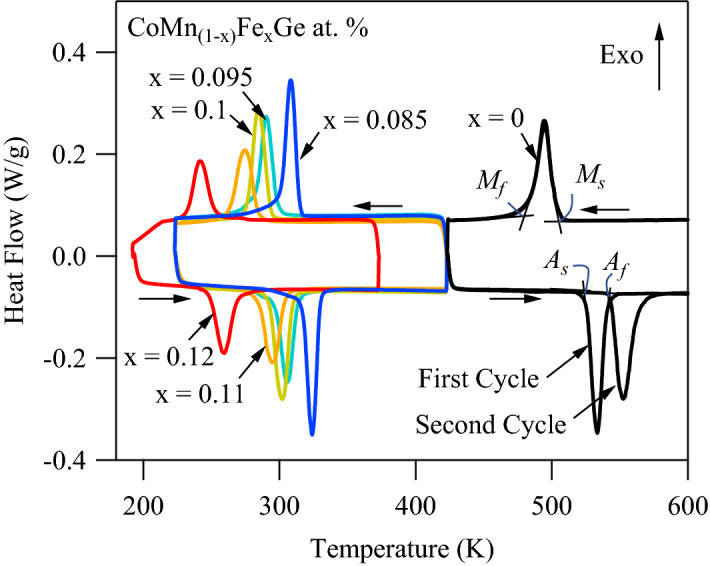
Thermograms of CoMn_1-x_Fe_x_Ge alloys (x = 0, 0.085, 0.0975, 0.1, 0.11, and 0.12) measured with differential scanning calorimetry (DSC) at 10 K min^−1^. Thermograms from two subsequent cycles are shown for pure CoMnGe, demonstrating the instability that may result from release of internal pressure in bulk polycrystals after the initial transformation decomposes the compound into powder. The transformation entropy change, $\Delta S$, was computed from the hexagonal to orthorhombic transition latent heat as 45.9 J kgK^−1^, 43.4 J kgK^−1^, 42.2 J kgK^−1^, 41.5 J kgK^−1^, and 40 J kgK^−1^ for the x = 0.085, 0.0975, 0.1, 0.11, and 0.12 samples, respectively.

Figure 2 illustrates the DSC thermograms of pure CoMnGe and CoMn_1-x_Fe_x_Ge (x = 0.085–0.12) at. % bulk polycrystals. The martensitic transformation temperatures are labeled in the figure for pure CoMnGe. Here the transformation temperatures are denoted ${A}_{f}$, ${A}_{s}$, ${M}_{s}$, and ${M}_{f}$ for the austenite finish, austenite start, martensite start, and martensite finish, respectively.

In the pure CoMnGe compound, two cycles are depicted to demonstrate the instability observed in the transformation temperatures. In this alloy, the thermal hysteresis (${A}_{f}-{M}_{s}$) was initially smaller, and on subsequent cycling, the hysteresis increased by nearly 20 K. It is important to note that the annealed CoMnGe compound began testing at room temperature in its bulk polycrystalline state from uncontrolled cooling, and was heated to a starting test temperature of 420 K. After multiple thermal cycles across the magnetostructural transition, the sample was removed from the DSC in powder form suggesting that the compound released internal pressure from the martensite to austenite transformation.

Increasing the Fe content resulted in a non-monotonic decrease in the martensite transformation temperatures^54^ and an increase in latent heat peak width as shown in the figure. The entropy change across the martensitic transformation was computed for each composition using the typical expression, $\Delta S=\frac{\Delta {H}_{latent}}{{T}_{m}}$, where $\Delta {H}_{latent}$ is the latent heat and ${T}_{m}$ is the average martensitic transformation temperature (${M}_{s}+{M}_{f}$)/2. Like the decrement in the transformation temperatures, the $\Delta S$ was also observed to slightly decrease from 45.9 J kgK^−1^ for x = 0.085 to 40 J kgK^−1^ for x = 0.12. Interestingly, no secondary ($\lambda$) peaks were observed above or below the first-order transition peak, which are typically indicative of magnetic transitions in DSC, such as the ferromagnetic Curie point.

### Thermo-magnetic transitions

**Figure 3:**
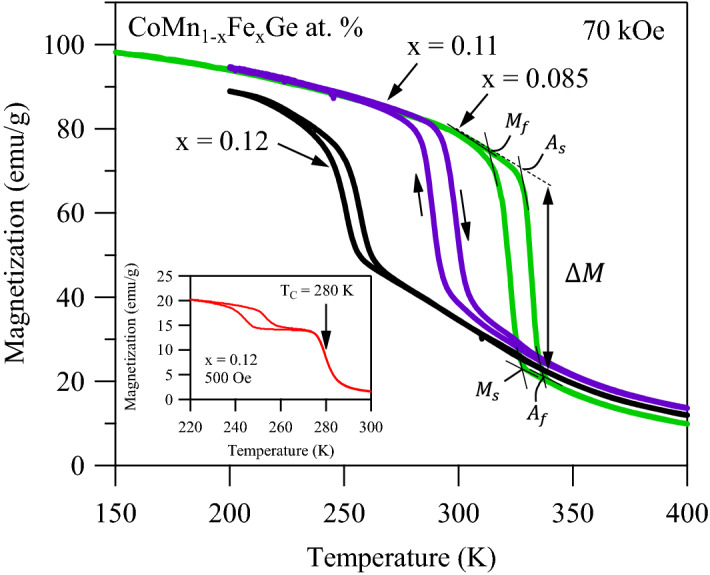
Thermomagnetic meta-magnetic behavior in CoMn_1-x_Fe_x_Ge (x = 0.085, 0.11, and 0.12) at. % alloys under 70 kOe applied magnetic field. The magnetization change, $\Delta M$, was measured to be 55.5 emu g^−1^, 41.6 emu g^−1^, and 25.2 emu g^−1^ for the x = 0.085, 0.11, and 0.12 at. % alloys, respectively. The thermomagnetic response under 500 Oe of x = 0.12 was depicted in the inset. The ferromagnetic Curie point of the austenite phase was clearly observed at 280 K away the magnetostructural transition indicating that replacing Mn with Fe in excess of x = 0.12 at. % decreased the entropy change and MCE.

Since one goal of the present work was to quantify the giant MCE in CoMnGeFe compounds, thermo-magnetic (MT) measurements were performed on x = 0.085, 0.11, and 0.12 as described in the experimental details section. Figure 3 depicts the MT curves under 70 kOe across the magnetostructural transition in which each compound exhibited a magnetization change, $\Delta M$, and thermal hysteresis. Transformation temperatures measured through MT nearly match those measured with DSC measurements in Fig. 2, suggesting that transformation temperatures have a relatively low sensitivity to the applied magnetic field and small CC slopes, mentioned earlier. Interestingly, alloys with less Fe exhibited a larger $\Delta M$ and a larger thermal hysteresis. However, replacing Mn with Fe is shown to be effective at decreasing the transformation temperatures of pure CoMnGe to near room-temperature for MCE applications and preserve the large $\Delta M$ at the concurrent magnetic and structural transition temperatures.

In the inset of Fig. 3, the MT response of x = 0.12 is depicted under 500 Oe. At this low field level, the martensitic transformation temperatures are clearly below the ferromagnetic Curie point of the austenite phase ($\approx 280 K$). Magnetization data demonstrates that substituting Fe for Mn in excess of 0.12 at. % can be expected to decouple the martensitic transformation from the Curie point of austenite, thus resulting in a decrease in entropy change as evidenced by the DSC data shown in Fig. 2.

**Figure 4:**
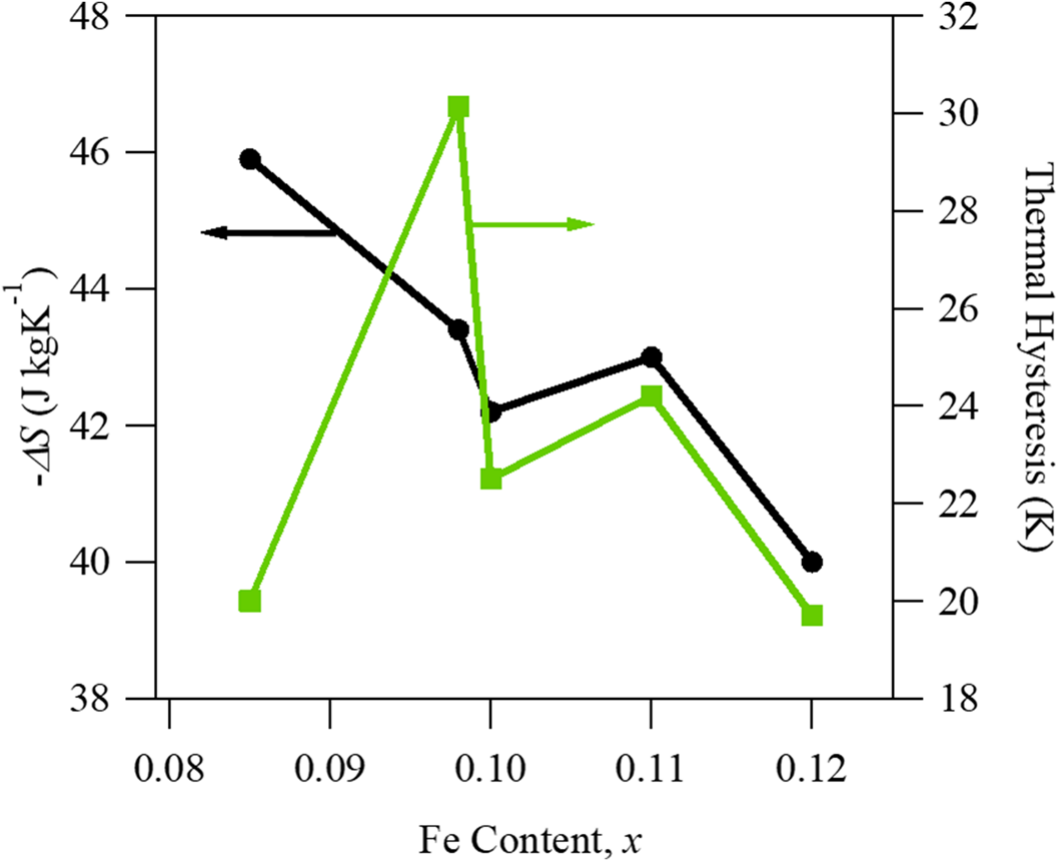
Transformation entropy change ($\Delta S$) and thermal hysteresis as a function of Fe content in CoMn_1-x_Fe_x_Ge at. % (x = 0.085, 0.0975, 0.1, 0.11, 0.12) alloys. Clausius-Clapeyron slopes, i.e. transformation temperature-magnetic field sensitivities, were measured to be 1.54 K T^−1^, 0.84 K T^−1^, 1.25 K T^−1^, 1.14 K T^−1^, 0.88 K T^−1^, in the x = 0.085, 0.0975, 0.1, 0.11, and 0.12 alloys, respectively.

For a better comparison between the compounds, the $\Delta S$ and thermal hysteresis was plotted as a function of Fe content in Fig. 4. The CC slopes from MT data, like that shown in Fig. 3, are denoted in the figure caption for each composition. It is clear from Fig. 4 that the lower Fe content resulted in the largest potential $\Delta S$, and thus giant MCE around room temperature. Interestingly, the compounds in the spread that contain the highest and lowest percentage of Fe exhibited nearly equal thermal hysteresis of 20 K. It should be noted that thermal hysteresis is not exclusively dependent on composition but has also been shown to be related to microstructural features and residual stress generated via excessive post-anneal cooling rates^56^. Naturally, there are errors associated in hysteresis measurements in all reported data and the present case is no exception. Nonetheless, the x = 0.085 specimen exhibited the largest $\Delta M$ of 55.5 emu g^−1^ at the magnetostructural transition under 70 kOe (Fig. 3), the highest Clausius-Clapeyron slope ($\approx$ 1.54 K T^−1^), and thus the largest $\Delta S$. Moreover, the thermal hysteresis was among the lowest in the composition spread, therefore the x = 0.085 compound was selected for further testing.

### Thermo-magnetic transitions in CoMn_0.915_Fe_0.085_Ge

**Figure 5:**
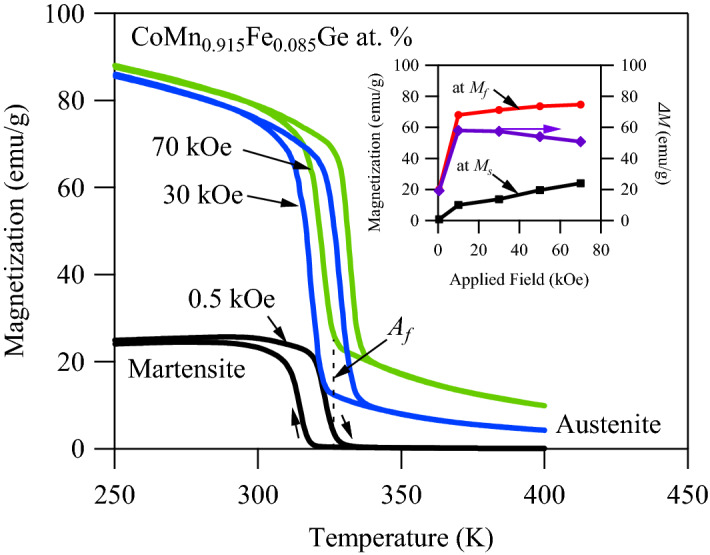
Thermo-magnetization curves for CoMn_0.915_Fe_0.085_Ge at. % alloy under constant magnetic fields of 0.5 kOe, 30 kOe, and 70 kOe. Curves were obtained via field heating (FH) and field cooling (FC) protocols with a temperature ramping rate of 5 K min^−1^. The inset depicts magnetization levels at ${M}_{f}$, ${M}_{s}$ , and their difference, $\Delta M$, as a function of applied field, $H$.

Figure 5 illustrates the MT curves of x = 0.085 under 500 Oe, 30 kOe, and 70 kOe from FC and FH experiments described, above. The orthorhombic martensite phase was found to be ferromagnetic and saturate with the application of nearly 10 kOe. As higher magnetic fields were applied, the martensitic transformation temperatures increased. This was one key difference between CoMnGeFe compounds and conventional NiMn-based MMSMA^76^ mentioned earlier. The x = 0.085 specimen exhibited an increase in transformation temperatures at a rate 1.54 K T^−1^, i.e. the CC slope. The inset depicts the magnetization levels of the martensite and austenite phases at the beginning (${M}_{s}$) and end (${M}_{f}$) of the forward, austenite-to-martensite transformation. As expected, the saturation magnetization of martensite at ${M}_{f}$ did not significantly increase beyond 80 emu g^−1^ with fields in excess of 10 kOe. Conversely, the austenite phase exhibited a significant and continual increase in magnetization at the ${M}_{s}$ temperature from 0.1 emu g^−1^ at 0.5 kOe to above 20 emu g^−1^ at 70 kOe, thus the magnetization change, $\Delta M$, from austenite to martensite initially increased until martensite was magnetically saturated, but then gradually decreased as austenite was continually magnetized with higher field levels. Using the CC equation mentioned above, with the MT data provided in Fig. 5, the maximum $\Delta S$ of the x = 0.085 compound was expected to be around 39 J kgK^−1^, which nearly matched that measured via the latent heat with calorimetry.

Typically, the CC slope in MMSMAs can range from $\pm$ 0.5 K T^−1^ to $\pm$ 6 K T^−1^^28,52,65,77–79^ and, therefore, in CoMnGeFe it is comparatively low. This parameter not only influences the $\Delta S$ through the CC equation, but also the attainability of the $\Delta S$ through magnetic field-driven transformations^67^. In application, the $\Delta S$ in the CoMnGeFe compound can be produced by pinning the material’s temperature at ${A}_{f}$, observed in Fig. 5 under 0.5 kOe by a vertical dashed line, then ramping the magnetic field to shift the transformation temperatures relative to the initial ${A}_{f}$. While holding the compound’s temperature constant at ${A}_{f}$ and ramping a magnetic field to 70 kOe (and subsequently increasing the transformation temperatures relative to the initial ${A}_{f}$), it is clearly seen in Fig. 5 that martensite will not saturate and, thus, only a small portion of the $\Delta S$ from the magnetostructural transformation will be produced.

In terms of measuring the giant MCE in CoMnGeFe compounds, a small CC slope implicitly defines a small temperature window in which isothermal magnetization measurements need to be performed to construct $\Delta S-T$ diagrams with Eq. (1). In Eq. (1), the $\Delta S$ is defined by the difference between integrals of isothermal (de)magnetization curves divided by the temperature difference between their measurements. The $\Delta S$ computed with this method has been shown to be sensitive to the size of the temperature increment between isothermal magnetization loops^67^, whereby the $\Delta S$ can be significantly overapproximated if the temperature increment is too large. An acceptable temperature increment, $\Delta {T}_{k}$, that would yield accurate results depends on both the CC slope and the sharpness of the transformation interval, e.g. ${A}_{f}-{A}_{s}$ of the compound in question. In x = 0.085, the experimental temperature increment was originally selected to be 3 K and isothermal measurements were performed between the highest and lowest ${A}_{f}$ temperatures shown in Fig. 5 to construct the $\Delta S-T$ diagrams.

### Isothermal magnetization loops in CoMn_0.915_Fe_0.085_Ge at. %

**Figure 6:**
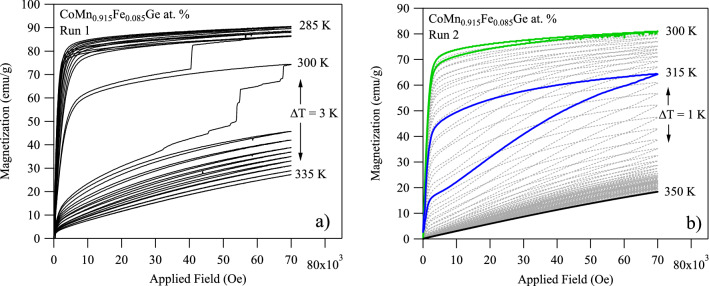
Isothermal magnetization loops in virgin CoMn_0.915_Fe_0.085_Ge at. % compounds up to 70 kOe with a 3 K temperature increment between measurements (**a**) and isothermal magnetization loops in pre-cycled powder with a 1 K temperature increment (**b**).

Figure 6a,b depict the typical magnetization, MH, loops for x = 0.085 in the vicinity of the martensitic transformation temperatures. At lower temperatures, a higher saturation magnetization was reached because more of the martensite phase had magnetized. In Fig. 6a, the austenite magnetization level increased from nearly zero to 25 emu g^−1^ at 335 K when 70 kOe was applied. Additionally, at 300 K a large hysteresis loop was measured indicative of the losses produced by a magnetic field-driven martensitic transformation. In this case, the magnetization loop exhibited two unexpected features, including (1) subsequent abrupt changes in magnetization around 50 kOe and 70 kOe, suggesting a burst-type martensitic transition had occurred, and (2) a magnetic field-induced austenite to martensite phase transition at 300 K, i.e. nearly 20 K lower than that measured in DSC.

As mentioned in the experimental section, the first MH run, depicted in Fig. 6a, was performed on a virgin bulk specimen that may have exhibited lower transformation temperatures compared to the DSC-powdered specimen due to the uncontrolled cooling rate from annealing. As mentioned above, hydrostatic pressure on (or within) CoMnGe compounds has been shown to cause a decrease in transformation temperatures. Reducing this internal pressure by controlling cooling rates has also been shown to increase the transformation temperatures. Moreover, the burst-type transformation that was observed via magnetization loops suggested rapid release of internal pressure at the point of the austenite to martensite phase transition. We believe that during the burst-type transformation, observed in Fig. 6a, the stored internal pressure in the austenite microstructure was rapidly released, thus transforming the x = 0.085 compound from a bulk polycrystal to a powder. Concurrently, the martensitic transformation temperatures should be expected to increase due to the release of the internal pressure.

Magnetization loops from the second MH run are depicted in Fig. 6b. To overcome errors in data post-processing introduced by the burst-type transformation depicted in Fig. 6a, the second MH run was performed in temperature increments of 1 K. The aim of decreasing the temperature interval was to better quantify the difference between the isothermal magnetization loops that are integrated in Eq. (1), as excessively large areas under the magnetization loops are depicted in Fig. 6a. On subsequent MH runs, however, the burst-type transformation was not observed. We believe this was because the internal pressure had already been released and the once bulk polycrystal had decomposed into a powder. Moreover, the magnetic field-induced austenite to martensite transformation exhibited a smaller magnetic hysteresis and, as expected, was observed at a higher transformation temperature (315 K). This behavior is congruent with the “virgin effect” reported elsewhere^69^.

### $\Delta S-T$ in CoMn_0.915_Fe_0.085_Ge at. %

**Figure 7:**
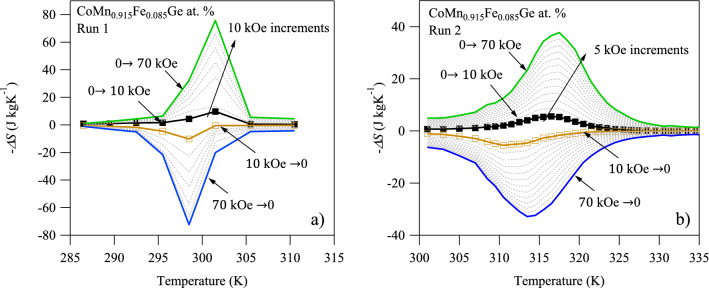
The maiden temperature dependence of (**a**) magnetic entropy change, $\Delta S-T$, in CoMn_0.915_Fe_0.085_Ge at. % bulk alloy with a 3 K temperature increment in Eq. (1) for applied field levels of 10, 20, 30, 40, 50, 60, and 70 kOe, and (**b**) the subsequent $\Delta S-T$ behavior after the bulk specimen had disintegrated into powder with a 1 K step measurement for applied field levels between 10 and 70 kOe in increments of 5 kOe.

Magnetization data in Fig. 6a,b and Eq. (1) were used to construct the magnetocaloric $\Delta S-T$ diagrams depicted in Fig. 7a,b for MH run 1 and run 2, respectively. As shown in Fig. 7a, post-processing data from Fig. 6a produced spuriously high $\Delta S$ that did not match DSC-computed entropy changes nor that computed using the CC equation, above. $\Delta S-T$ results were unrealistically high as a result of the burst-type transformation that produced large areas under the magnetization loops with Eq. (1). Apparently, the transformation was too abrupt in the annealed bulk polycrystals and 3 K temperature increments were too large to produce reliable $\Delta S-T$ diagrams. Moreover, the maximum $\Delta S$ ($\approx$ 80 J* kgK*^−1^) in Fig. 7a is observed at 300 K which is nearly 20 K less than expected from transformation temperatures in DSC measurements.

Finally, the $\Delta S-T$ diagrams constructed using MH data from run 2 are depicted in Fig. 7b. Due to the absence of the burst-like transformation and the smaller $\Delta {T}_{k}$, the $\Delta S-T$ diagram was smooth and provided a reasonable approximation of $\Delta S$ over a temperature interval that nearly matched those measured under zero magnetic field in DSC. Moreover, it was apparent that the magnetic field did not significantly influence the latent heat of the martensitic transformation in CoMnGeFe up to 70 kOe.

Clearly, CoMnFeGe compounds exhibited a cyclic transformation instability due to the pressure dependence of the transformation temperature and a release of internal hydrostatic pressure during disintegration (transformation). If the bulk material was cooled at an uncontrolled rate post-annealing, or following arc-melting, the internal hydrostatic pressure may produce lower-than-expected transformation temperatures (here, we observed 20 K lower). Upon cycling the compound from the austenite to martensite phase by magnetic field ramping, the bulk material disintegrated into a powder, thus releasing internal pressure, increasing the transformation temperatures, and revealing the interesting behavior of the virgin-effect.

The pressure-transformation temperature sensitivity of the CoMnFeGe compound and its excessive brittleness may be viewed as detrimental in terms of regenerator applications, yet powder bed systems are widely accepted as a viable method of cooling. Powder bed regenerators are currently under scrupulous investigation by numerous groups^80–84^. Therefore, cycled CoMnGe compounds may still be a contender for caloric applications. From a regenerator design perspective, any studies that report on critical transformation temperatures in bulk CoMnGe compounds, from maiden transformation cycles, should be critically interpreted to avoid misleading results. Multiple thermal, or transformation, cycles are required to fully characterize its MCE due to its pressure dependence.

## Concluding remarks

Transformation temperatures and the magnitude of the magnetocaloric entropy change across the hexagonal to orthorhombic martensitic transformations in CoMn_1-x_Fe_x_Ge (x = 0, 0.085, 0.0975, 0.1, 0.11, and 0.12) were observed to exhibit a pressure dependence that diminished after maiden transformation cycles. Bulk specimen that were arc-melted and annealed were found to disintegrate into powder following thermal and magnetic field cycling. The release of internal pressure from the disintegration process increased the transformation temperatures by nearly 20 K and enabled the accurate quantification of the temperature dependent magnetocaloric effect ($\Delta S-T$) using the discretized Maxwell relationship.
